# The Proactive-Reactive Resilience as a Mediational Variable Between the Character Strength and the Flourishing in Undergraduate Students

**DOI:** 10.3389/fpsyg.2022.856558

**Published:** 2022-05-18

**Authors:** Jesús de la Fuente, Begoña Urien, Elkin O. Luis, María Carmen González-Torres, Raquel Artuch-Garde, Alvaro Balaguer

**Affiliations:** ^1^School of Education and Psychology, University of Navarra, Pamplona, Spain; ^2^School of Psychology, University of Almería, Almería, Spain; ^3^School of Health and Psychology, Public University of Navarra, Pamplona, Spain

**Keywords:** strength character, proactive and reactive resilience, flourishing, university, mediational model

## Abstract

The aim of this research was to delimit the predictive and mediational model of resilience between character strengths to predict flourishing, in a sample of undergraduate students. After signing their informed consent, 642 university students completed three validated scales (i.e., character strengths, resilience, and flourishing). Using an ex post facto design, regression, structural modeling, and mediation analyses were carried out, in order to construct a multi-causal predictive model. Results indicated a consistent predictive direct effect of character strengths on resilience and flourishing and of resilience on flourishing. As hypothesized, resilience also showed a mediating effect on the relationship between character strengths and flourishing. Additionally, results also revealed that the reactive and proactive factors of resilience were explained by different character strengths (e.g., emotional strength/cognitive, interpersonal strengths), reinforcing the idea that the two directions are complementary and necessary. Finally, several implications were established for the practice of positive psychology.

## Introduction

Positive Psychology (PP) focuses on factors that promote human wellbeing, in contrast to the classic study of factors involved in disorders (Joseph and Linley, 2005), and it is defined as the scientific study of what makes life most worth living (Seligman and Csikszentmihalyi, 2000; Ghielen et al., 2018). Thus, positive psychology ultimately seeks to explain and predict psychological wellbeing (Dodge et al., 2012). In this regard, a wellbeing model based on actual positive psychology (PP 2.0) needs to enhance individuals’ positive traits, attitudes, and behaviors and/or manage negative ones in order to promote wellbeing and decrease mental illness at the same time (Ivtzan et al., 2016). PP 2.0 recognizes that wellbeing involves a dialectical balance between the “light” and the “dark” aspects of life (Lomas, 2016).

The PP, has also influenced the emergence and interest by of new psychological constructs (Duckworth et al., 2005; Proyer et al., 2013; Ciarrochi et al., 2016) such as character strengths, subjective wellbeing, psychological wellbeing, hedonic wellbeing, eudaimonic, flourishing, happiness, thriving or resilience which have become part of this new psychological dictionary (Dodge et al., 2012; Ruch and Hofmann, 2017; Schreiner, 2018).

The present paper adopts flourishing as an integrated approach to study wellbeing, which includes both hedonic and eudaimonic wellbeing traditions (Patnaik, 2021). Flourishing/Flowering defined as the favorable relationship between individuals, their environment, and culture, which in turn influences the experimentation of high levels of wellbeing and consequently of mental health have developed an important body of research (Keyes, 2002).

Although character strengths (i.e., positive stable personality traits; Wagner et al., 2020), resilience (i.e., the ability to resist negative events and/or to recover from them; Harms et al., 2018), and flourishing have emerged from different research domains, they share the common denominator of promoting psychological wellbeing, while they are related to each other in ways yet to be defined (Donaldson et al., 2015; Ciocanel et al., 2017). In addition to flourishing, character strengths and resilience are also complex constructs. Character strengths are organized under six broad virtues: (1) Wisdom and knowledge; (2) Courage; (3) Humanity; (4) Justice; (5) Temperance; and (6) Transcendence (Peterson and Seligman, 2004). On the other hand, resilience in recent studies suggests that it consists of two dimensions: reactive and proactive factors (de la Fuente et al., 2021a).

Thus, the present paper seeks to clarify the relationships between the mentioned variables by answering the following questions: Do character strengths predict resilience? To what degree do these two predict flourishing? To what extent do character strengths and resilience contribute to the flourishing of under-graduate students? From a research perspective, our work will contribute to establishing which character strengths relate to resilience, to confirming whether resilience comprises of two sub-dimensions, and to unfolding direct and indirect relationships among the variables under study. From an applied perspective, this paper could direct psychological interventions in order to improve flourishing in under-graduate students.

### Wellbeing and Flourishing

Two different conceptual frameworks usually explain wellbeing (Chaves, 2021). The *hedonic paradigm* (*subjective wellbeing as* defined as by Diener’s model) considers wellbeing as the result of the cognitive and affective evaluation that a person makes of his life, which leads him to experience high levels of positive affect, low levels of negative affect, high life satisfaction, and happiness (Diener, 2009). For its part, the *eudaimonic paradigm* (*psychological wellbeing* as defined by Ryff’s model) understands wellbeing as human potential and highlight that wellbeing is facilitated by overcoming life challenges through applying human potential traits. He proposes six key components of wellbeing: purpose in life, mastery of the environment, positive relationships, self-acceptance, personal growth, and autonomy) (Ryff and Singer, 2006; Ryff, 2014, 2016). Since both perspectives can complement each other, tentative attempts have been proposed in order to unify these two traditions (Hedonistic and Eudaimonic), with the aim of comprehensively explaining wellbeing. In fact, mental wellbeing is currently understood as the combination between the hedonic and eudaimonic perspectives (Ryan and Deci, 2001; Schotanus-Dijkstra et al., 2016; Chaves, 2021). Recent definitions of flourishing combine hedonic and eudaimonic elements to create a more comprehensive and holistic approach (Chaves, 2021).

From a temporal perspective, initially, the most influential perspective on human flourishing was the eudaimonic (VanderWeele, 2020). However, from a psychological perspective, flourishing also incorporates the hedonic experience (Schotanus-Dijkstra et al., 2016). In this respect, extant research (Keyes, 2007), as well as recent studies, also show the importance of improving flourishing so as to increase hedonic wellbeing (Allison et al., 2020; Lee et al., 2021).

Thus, these efforts lead to jointly considering both perspectives (hedonic and eudaimonic) of wellbeing. That is, to identify positive psychological states (subjective wellbeing) together with the value and impact of hedonic experiences that motivate and reinforce the search for basic needs satisfaction, and the update of human potential (significant vocation and a sense of purpose). Based on the above-mentioned, the present paper’s approach to wellbeing follows this integrative perspective and accepts that flourishing comprises both wellbeing traditions.

These integrated eudaimonic and hedonic perspectives are based on influential theoretical approaches. Firstly, the model of human potential (Ryff and Singer, 2006), which encompasses emotional and physical health, evaluates autonomy, personal growth, self-acceptance, life purpose, mastery, and positive relationships. Secondly, self-determination theory (Ryan and Deci, 2000), considers that “self-realization” is a facilitator of wellbeing experience, and emphasizes the fundamental role that autonomy, competence, and relationships play in flourishing. Finally, Seligman’s (2018) PERMA model, includes five fundamental aspects for human flourishing. These aspects are the experience of positive emotions, personal commitment to what is done, the establishment of positive interpersonal relationships, search for meaning, meaning, and purpose in life, and agency capacity. More recently, from a more interactive and contextual approach, the Self-vs. External-Regulation Theory, SR-ER model (de la Fuente et al., 2017; de la Fuente-Arias, 2017) has proposed that the achievement of psychological wellbeing is a consequence of the combination of the optimal levels of personal and contextual regulation.

Thus, Flourishing is a psychological construct referring to the experience that life is going well which combines a sense of feeling good and effective functioning. Flourishing is considered a personal indicator that corresponds to a high level of mental wellbeing (Huppert, 2009a,b). It is a global construct that integrates psychological variables from the hedonic and eudaimonic perspectives (e.g., perceived competence, emotional stability, engagement, meaning, optimism, positive emotions, positive relations, self-esteem, and vitality (Huppert and So, 2013).

### Character Strengths and Flourishing

Character strengths spring from the search for personal values that make us strong and able to face life and adversity (Park et al., 2004; Lavy, 2019; Karris-Bachik et al., 2020). Character strengths may be considered the psychological ingredients of virtues (Park et al., 2006). Based on this construct, the Values in Action Inventory of Strengths (VIA-IS; Park et al., 2004) was designed and it was also adapted to educational settings (Berkowitz, 2002; Vargas and González-Torres, 2009). Character strength meets the following criteria: 1. Ubiquity—is widely recognized across cultures; 2. Fulfilling—contributes to individual fulfillment, satisfaction, and happiness broadly constructed; 3. Morally valued—is valued in its own right and not for tangible outcomes it may produce; 4. Do not diminish others—elevate others who witness it, producing admiration, not jealousy; 5. Non-felicitous opposite—has obvious antonyms that are “negative”; 6. Traitlike—is an individual difference with demonstrable generality and stability; 7. Measurable—has been successfully measured by researchers as an individual difference; 8. Distinctiveness—is not redundant (conceptually or empirically) with other character strengths; 9. Paragons—is strikingly embodied in some individuals; 10. Prodigies—is precociously shown by some children or youth. 11. Selective absence—is missing altogether in some individuals; 12. Institutions—are the deliberate target of societal practices and rituals that try to cultivate them (Park et al., 2004).

Based on our characterization of flourishing which includes both hedonic and eudemonic wellbeing (Huppert and So, 2013), extant research broadly reports significant relationships between character strengths and the former (Brdar and Kashdan, 2010; Buschor et al., 2013; Hausler et al., 2017; Athota et al., 2020; Baumann et al., 2020; Villacís et al., 2021). Additionally, specific research which addresses wellbeing as flourishing confirms the positive relationship between both variables (Baumann et al., 2020; Niemiec, 2020). Further support comes from multicomponent interventions (Hendriks et al., 2019), which report flourishing amelioration via improving character strengths (VanderWeele, 2020). Along the same lines, extant research reports several experiences focus on improving both character strengths and flourishing (VanderWeele, 2017a,b, 2020; Schutte and Malouff, 2019). Similar results are found when studying the impact of character strengths on thriving as part of flourishing (Hausler et al., 2017).

Another line of studies analyses character strengths structural characteristics and their effects on flourishing (Heintz and Ruch, 2020). These studies report that the strongest cross-sectional associations of flourishing are with hope, curiosity, love, and gratitude (Emmons and Stern, 2013). Although extant longitudinal research reports that character strengths tend to be stable over time (Emmons and Stern, 2013; Wagner et al., 2020), other studies found that character strengths such as humor, spirituality, and prudence may be more susceptible to change (Wagner et al., 2020).

From the above-mentioned, a more in-depth analysis is necessary in order to specify which character strengths relate to flourishing as well as the strength of such a relationship.

### Resilience and Flourishing

The role of resilience, whether in protecting against stress, or in contributing to flourishing, has been conceptualized from several perspectives (e.g., rising above, adaptation, and adjustment; Aburn et al., 2016). Literature broadly supports the key role of resilience in coping with difficulties and in helping individuals to persist while pursuing their goals (Kim et al., 2016; Kachel et al., 2020). Additionally, the latest studies propose biased affective forecasting as a potential mechanism that promotes resilience and flourishing (Colombo et al., 2020).

Extant research confirms the positive relationship between resilience and flourishing (Yildirim and Belen, 2019). Additionally, recent studies analyze its value in personal recovery after health accidents (Rapport et al., 2020), in preventing psychopathological symptoms (Chmitorz et al., 2018), in mental health (Wu et al., 2020), or as a mediator between optimism and subjective wellbeing (He et al., 2013; Miranda and Cruz, 2020). However, a recent meta-analysis on resilience and flourishing dimensions shows that effect sizes among studies are heterogeneous, which points to a large variability within the reported results (Liu et al., 2020).

To further analyze resilience and based on the Connor-Davidson Resilience Scale (CD-RISC scale), recent research (de la Fuente et al., 2021a), suggests that resilience consists of two dimensions, that is, reactive and proactive. Reactive resilience comprises behavioral factors pertaining to endurance under adverse conditions (reactive factors). On the other hand, proactive resilience refers to the ability to bounce back and produce changes under unfavorable situations (proactive factors). Reactive resilience, stresses tolerance skills, and spirituality behaviors to predict emotion-focused coping strategies (stress, spirituality). Likewise, proactive resilience, i.e., the ability to adapt to change and perceived control (tenacity, control, change, spirituality), predict problem-focused strategies (de la Fuente et al., 2021a). Consequently, the two dimensions are complementary and necessary, although only the proactive factors could pertain to self-regulatory behavior (de la Fuente et al., 2021a). Additionally, the moderator role of this two-component model has also been tested between individual factors (big five) and the stress experience (de la Fuente et al., 2021b).

Thus, the present paper delves into the distinctive links between resilience dimensions (tenacity, control, change, stress, and spirituality) and flourishing in order to uncover the nuances of this relationship.

### Character Strengths and Resilience

Extant studies have already found positive relationships between character strengths and resilience (Chung, 2008; Hausler et al., 2017). Specifically, character strengths show predictive power beyond other related factors (i.e., positive affect, self-efficacy, optimism, social support, self-esteem, life satisfaction) as well as sociodemographic variables (Martínez-Martí and Ruch, 2017).

More recent research with US university students reports a consistent relationship between VIA character strengths and happiness, wellbeing, resilience, academic success, and psychopathologies (Karris-Bachik et al., 2020). Specifically, three functions of character strengths are highlighted when facing adversity: buffering (i.e., the use of character strengths prevents problems); reappraisal (i.e., a person’s character strengths explain or reinterpret problems); and resilience (i.e., character strengths support the bounce-back from life’s setbacks (Colombo et al., 2020).

Despite the foregoing evidence, relations between character strengths and resilience are neither sufficiently established, nor do we understand precisely how these two constructs are related to flourishing. Nevertheless, it seems reasonable to assume that character strengths are foundational to resilient behaviors and to flourishing itself.

### Aims and Hypothesis

**FIGURE 1:**
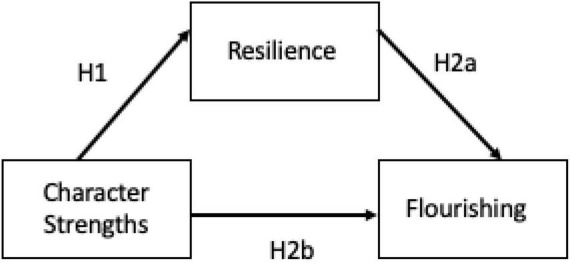
Study hypothesis. Hypothesis 1 (H1) seeks to test whether the constituent factors of character strengths positively predict resilience scores while hypothesis 2 (H2a and H2b) analyses which scores in character strengths (H2a) and resilience (H2b) predict (directly and indirectly) total scores in flourishing.

Consequently, the objectives of the present paper are: (a) to establish predictive relationships between character strengths and resilience; and (b) to determine the joint contribution of these two variables to predict flourishing. Accordingly, *hypothesis 1* (H1) seeks to test whether the constituent factors of character strengths positively predict resilience scores while *hypothesis 2* (H2) analyses which scores in character strengths and resilience predict (directly and indirectly) total scores in flourishing (H2a). Specifically, resilience may be expected to play a mediating role on flourishing with a direct and indirect effect (H2b) (Figure 1).

## Materials and Methods

### Participants

A convenience sample of 642 undergraduate students was formed from the two universities involved in this research project; these students subsequently completed questionnaires that assessed ten teaching-learning processes (i.e., ten academic subjects). The participants were enrolled in either Psychology or Primary Education degrees; 83.5% were female, and 16.5% were male. Mean age was 20.13 years (σ_χ_ = 5.8), and age range was 19–45 years. Participation was anonymous and voluntary. Lecturers from various departments were invited to participate, and those who agreed then extended the invitation to their students. Participating lecturers and students were awarded a Certificate of Participation. Online questionnaires were applied to assess each specific teaching-learning process.

### Instruments

#### The VIA Inventory of Strengths

The short form of the VIA Inventory of Strengths (VIA-IS-72) (Peterson and Seligman, 2004) includes 72 items, 24 factors and 6 dimensions, that allow subjects to self-assess on 24-character strengths. For each character strength, there are three Likert-style items with five possible responses, where 1 = “Very Much Unlike Me” and 5 = “Very Much Like Me.” For example, the statements include “I am a highly disciplined person” (self-regulation). According to measures of internal consistency reliability and validity, the VIA-72 is substantially equivalent to the original, long VIA-IS, as verified by its developers (VIA Institute on Character, 2020). VIA-72 psychometric report showed a mean Cronbach alpha (reliability) of 0.75 for the 24 scales, and Omega index of 0.71; coefficients ranged from 0.70 to 0.87 (VIA Institute on Character, 2020). The Spanish version of the VIA-72 was obtained for this study [CFI = 0.96, TLI = 0.94, RMSEA = 0.05, and SRMR = 0.06].

#### The Connor-Davidson Resilience Scale

The validated, Spanish version (Manzano-García and Ayala-Calvo, 2013) of the CD-RISC Scale (Connor and Davidson, 2003) was applied to measure resilience. Reliability and validity values are adequate in Spanish samples, and there is a five-factor structure: F1: Persistence/tenacity and strong sense of self-efficacy (*tenacity*); F2: Emotional and cognitive control under pressure (*stress*); F3: Adaptability/ability to bounce back (*change*); F4: Perceived Control (*control*), and F5, Spirituality (*spirituality*). Subjects are asked to rate statements such as “I give my best effort, no matter what the outcome may be” or “I believe I can achieve my goals, even if there are obstacles”. This Likert-type scale contains 25 items and five factors: (1) personal competence, high standards and *tenacity* (α = 0.80), (2) self-confidence, tolerance of negative affect and strengthening effects of *stress* (α = 0.75), (3) positive acceptance of *change*, and secure relationships (α = 0.77), (4) *control* (α = 0.71), and (5) *spiritual* influences (0.71) and an Omega index of.76. Adequate reliability and validity values had been obtained in Spanish samples, and a five-factor structure emerged [Chi-square = 1,619, 170; Degrees of freedom (350–850) = 265; *p < 0*.001;Ch/Df = 6,110; SRMR (Standardized Root Mean-Square) = 0.062; NFI (Normed Fit Index) = 0.957; RFI (Relative Fix Index) = 0.948; IFI (Incremental Fix Index) = 0.922; TLI (Tucker Lewis index) = 0.980; CFI (Comparative fit index) = 0.920; RMSEA (Root Mean Square Error) = 0.063; HOELTER = 240 (*p* < 0.05) and 254 (*p* < 0.01)].

#### The Flourishing Scale

A Spanish version validated in Spanish populations (Garzón-Umerenkova, 2016; Pozo-Muñoz et al., 2016; Garzón-Umerenkova et al., 2017) of the Flourishing Scale (Biswas-Diener et al., 2009) was applied. This scale seeks to measure flow or flow state (flourishing) and contains eight items on a five-point Likert scale. Responses range from 1 (strongly disagree) to 5 (strongly agree). Subjects are asked to rate statements such as “I have a useful and meaningful life” or “I am a good person and I live a good life”. Cronbach alpha for the Spanish sample is 0.85; and an Omega index of 0.81. The unidimensionality of the scale and the metric invariance in the evaluated samples was confirmed [Chi-square = 79,392; Degrees of freedom (44–24) = 20; *p* < 0.001;Ch/Df = 3,970; SRMR (Standardized Root Mean-Square) = 0.052; NFI (Normed Fit Index) = 0.946; RFI (Relative Fix Index) = 0.953; IFI (Incremental Fix Index) = 0.959; TLI (Tucker Lewis index) = 0.955; CFI (Comparative fit index) = 0.958; RMSEA (Root Mean Square Error) = 0.039; HOELTER = 757 (*p* < 0.05) and 905 (*p* < 0.01)].

### Procedure

University students voluntarily completed the validated questionnaires, after signing their informed consent via the online platform (de la Fuente et al., 2015). Five specific teaching-learning processes were assessed, corresponding to different academic subjects that were taken over a 2-year period. In September-October of 2018 and 2019, presage variable (characters strength) were assessed. Process variable (resilience) were measured in February-March of 2019 and 2020, and product variable (flourishing) in May-June of 2019 and 2020. Approval for this procedure was obtained from the Ethics Committee of University of Navarra, within a larger R&D Project (2018–2021; ref. 2018.170).

### Data Analysis

An *ex post facto prospective and* transversal design (Lohr, 1999) was used. The *ex post facto* design come to solve the problem that occurs when the variable of interest has already occurred and/or it is not ethical to cause it. It is *prospective* because the independent variable precedes the dependent variable in the analysis. Additionally, it is cross-sectional because longitudinal follow-up is not possible and the data was collected within a short period of time. Three types of analyses were performed. The usual assumptions of regression analysis were tested beforehand.

*(1) Preliminary analysis.* First, the quality of the data was explored by testing for outliers and missing cases. *Univariate* outliers were tested by calculating the typical scores of each variable, considering cases with Z scores outside the ±3 range to be potentially atypical cases (Tabachnick et al., 2007). On the other hand, the Mahalanobis distance (D2) was used to detect atypical *combinations* of variables (atypical multivariate cases), a statistical measure of an individual’s multidimensional distance from the centroid or mean of the given observations (Lohr, 1999). This procedure detects significant distances from the typical combinations or centroids of a set of variables. Literature suggests removing univariate and multivariate outliers, or reassigning them to the nearest extreme score (Weston and Gore, 2006). The procedure was carried out using SPSS (v.26, IBM, Armonk, NY, United States), which provides a specific routine for missing values analysis that determines the magnitude of missing values and whether they are presented in a systematic or random manner.

Assumptions related to sample size, independence of errors, univariate, and multivariate normality, linearity, multicollinearity, recursion, and interval measurement level were also evaluated, showing acceptable reliability levels. Regarding the sample size, inclusion of 10–20 cases per parameter is recommended, and at least 200 observations (Kline, 2005).

Independence of error means that the error term of each endogenous variable must not be correlated with other variables. In order to test for univariate normality, the distribution of each observed variable was examined, and its indices of asymmetry and kurtosis. Asymmetry values greater than 3 and kurtosis greater than 10 suggest that the data should be transformed. Additionally, values less than 70 on the Mardia multivariate index indicate that the distance from the multivariate normality is not a critical deterrent to this analysis (Mardia, 1970). Although one of the assumptions is the level of interval measurement, in some cases, variables measured at a nominal or ordinal level were used, as long as the distribution of scores, particularly of the dependent variables, were not markedly asymmetric.

*(2) Predictive analysis.* For Hypothesis 1, multiple regression analysis was applied using SPSS (v.25)

*(3) Structural prediction and mediational models.* Hypotheses *2* were tested using a Structural Equation Model (SEM) and a mediational model for complex measurement (Ato and Vallejo, 2011). Model fit was assessed by first examining the ratio of chi-square to degrees of freedom, then the Comparative Fit Index (CFI), Normed Fit Index (NFI), Incremental Fit Index (IFI), Relative Fit Index (RFI) and Tucker-Lewis Index (TLI). All fit measures of the incremental model were above the suggested limit of 0.90 (Bentler, 1990). The value of the Comparative fit index (CFI) was equal to 0.928, which is also satisfactory. The results of the original scale were replicated. The value of the Root Mean Square Error of Approximation (RMSEA) was 0.08 less than the warning value of 0.09 (Ho, 2006). Ideally, these should be greater than 0.90. The Hoelter Index was also used to determine the adequacy of sample size. AMOS (v.22) was used for these analyses. Keith (2006) proposed the following beta coefficients as research benchmarks for *direct effects*: less than 0.05 is considered too small to be meaningful, above 0.05 is small but meaningful, above 0.10 is moderate, and above 0.25 is large. For *indirect effects*, we used Kenny’s (2012) definition which considers them as the product of two effects; using Keith’s benchmarks above, we propose a small indirect effect = 0.003, moderate = 0.01, and large = 0.06, values that are significant in the sphere of education.

## Results

Results from the analyses applied in order to test normality, a prerequisite for linear analysis, show an adequate distribution of sample variability (see Table 1).

**TABLE 1:** Descriptive values of the variables under study (*n* = 641).

Variable	Min	Max	*M* (*SD*)	Mean std. error	Asymmetry	Standard asymmetry error	Kurtosis	Standard kurtosis error	Kolmogoroff-Sminoff
D1	1.93	5.00	3.655(0.595)	0.059	0.116	0.116	–0.303	0.343	0.065, *p* < 0.200
D2	1.80	5.00	3.823(0.551)	0.039	–0.105	0.175	0.044	0.348	0.056, *p <* 0.200
D3	2.74	5.00	4.000(0.530)	0.037	–0.205	0.174	–0.506	0.346	0.061, *p <* 0.200
D4	1.89	5.00	3.951(0.555)	0.040	–0.316	0.175	0.236	0.349	0.035, *p* < 0.200
D5	1.96	5.00	3.552(0.549)	0.038	0.233	0.170	0.070	0.377	0.064, *p <* 0.200
D6	2.10	5.00	3.578(0.603)	0.042	0.049	0.172	–0.281	0.343	0.043, *p* < 0.200
SCTOT	2.89	4.88	3.731(0.448)	0.038	0.355	0.206	–0.456	0.408	0.070, *p* < 0.200
Stress	1.43	5.00	3.627(0.539)	0.013	–0.162	0.061	–0.06	0.121	0.067, *p* < 0.200
Spirituality	1.00	5.00	3.257(1.00)	0.024	–0.071	0.060	–0.494	0.120	0.087, *p <* 0.155
Tenacity	1.00	5.00	3.912(0.591)	0.014	–0.450	0.060	0.286	0.121	0.074, *p <* 0.174
Change	1.63	5.00	3.942(0.613)	0.015	–0.489	0.060	0.462	0.120	0.058, *p <* 0.188
Control	1.20	5.00	3,893(0.755)	0.018	–0.516	0.060	0.499	0.120	0.69, *p <* 0.196
RESTOT	1.52	4.86	3.725(0.476)	0.121	–0.492	0.062	0.558	0.125	0.049, *p <* 0.200
FLUORISHING	2.13	5.00	4.100(0.629)	0.031	–0.569	0.121	0.015	0.241	0.045. *p <* 0.150

*D1, cognitive strength (Wisdom); D2, emotional strength (Courage); D3, interpersonal strength (Humanity); D4, Civic strength (Justice); D5, strengths that protect against excess (Temperance); D6, strength from the meaning of life (Transcendence). SCTOT, Total Strength Character; RESTOT, Total Resilience.*

### Predictive Value of Character Strengths on Resilience, and Flourishing

Regression analyses showed, that D2 (*emotional strength*) was the strength dimension that established significant relationships with four of the five resilience dimensions (*adaption to change*), total resilience and flourishing. Specifically, it was the strongest predictor of *stress tolerance*, *flourishing* and *tenacity*. D1 (*cognitive strength*) explained *resilience* strongly and *adaption to change*. D3 (*interpersonal strength*) was only related to *control* and D6 (*strength from the meaning of life*) predicted *resilience*. As expected, D5 (*strength against excess*) is negatively related to *control*. *Control* and *resilience* were the criteria variables predicted by more character strengths dimensions. So, *control* was explained by *emotional*, *interpersonal*, and *strength against excess* while *resilience* was predicted by *cognitive*, *emotional*, and *strength from the meaning of life*. Overall, *character strengths* explained 42% of the variance of *stress tolerance*, 33.7% of *tenacity* and 32.7% of *flourishing* (see Table 2).

**TABLE 2:** Multiple regression of the dimensions of character strengths, resilience and flourishing.

Strengths dimensions	Stress tolerance	Spiritual	Tenacity	Adaptation to change	Control	Total resilience	Flourishing
D1	0.193	0.110	0.028	0.295*	0.242	0.506***	0.028
D2	0.565***	0.344**	0.433*	0.215	0.340**	0.248**	0.443***
D3	0.003	0.137	0.087	0.117	0.276***	–0.108	0.087
D4	–0.034	–0.013	–0.110	–0.196	0.014	0.170	−0,110
D5	–0.161	–0.056	–0.116	–0.148	−0.266**	0.131	–0.116
D6	0.025	0.000	0.217	0.090	0.201	0.581***	0.035
*F*(6, 124) =	15,646***	7,185***	10,05***	15,646***	7,790***	4,501***	19,528***
*R* ^2^	0.421	0.249	0.337	0.208	0.262	0.170	0,327

D1, cognitive strength (Wisdom); D2, emotional strength (Courage); D3, interpersonal strength (Humanity); D4, Civic strength (Justice); D5, strengths that protect against excess (Temperance); D6, strength from the meaning of life (Transcendence). *p < 0.05; **p < 0.01; ***p < 0.001.

### Predictive Value of the Factors of Character Strengths on Resilience Factors and Flourishing

In more detail, multiple linear regression analyses showed that the factors that most significantly and positively predicted *total resilience* were F9 (*vitality and zest*), F6 (*bravery*) and F14 (*sense of justice*), and F19 (*self-regulation*) and F17 (*modesty, humility*), negatively. The most significant and negative predictors of *total flourishing* were F20 (*appreciation for beauty and excellence*) and F9, positively. Referring to specific factors of resilience, F9 and F7 (*perspective and diligence*) significantly predicted *tenacity*. *Stress tolerance* was positively predicted by factors F12 (*emotional intelligence*) and F9, and negatively predicted by F13 (*citizenship*). *Adaptation to change* was positively predicted by factors F12 and F15 (*leadership*), and negatively by F17, F18 (*prudence*), and F24 (*spirituality*). The resilience factor with the highest number of predictive character strengths was perceived *control*, being predicted by F9, F14, F10 (*love and be loved*), F2 (*love for knowledge*) and F7 (positively) and by F1 (*curiosity*), F11 (*kindness*), F20 (*appreciation for beauty*), F22 (*hope*) and F17 (negatively). Factors F21 (*gratitude*) and F19 were positive predictors of spirituality (F24), whereas F24 did not predict them (see Table 3).

**TABLE 3:** Multiple regression between the dimensions and factors of strengths, resilience and flourishing.

PV	Stress	Spirituality	Tenacity	Change	Control	Total resilience	Flourishing
**D1**							
F1					−0.378**		
F2					0.242*		
F3							
F4							
F5							
**D2**							
F6						0.214*	
F7			0.277*		0.173*		
F8							
F9	0.291*		0.399**		0.478***	0.409**	0.567***
**D3**							
F10					0.291**		
F11					−0.316*		
F12	0.343**			0.293*			
**D4**							
F13	−0.275*						
F14					0.314*	0.282*	
F15				0.239*			
**D5**							
F16							
F17				−0.194*	−0.174*	−0.157*	
F18				-0.185*			
F19		0.179*					
**D6**							
F20					−0.205*	−0.239*	−0.219*
F21		0.342**					
F22					−0.205*		
F23							
F24				−0.173*			
*F*	*F*(24,111) = 5.563***	*F*(24,114) = 2.961***	*F*(24,111) = 4,474***	*F*(24,112) = 2.709***	*F*(24,112) = 4.214***	*F*(24,106) = 4,336***	*F*(24,137) = 4,494***
*R* ^2^	0.208	0.384	0.492	0.367	0.475	0.497	0.512

**D1 = Cognitive strength (Wisdom):** F1, Curiosity, interest in the world; F2, Love for knowledge and learning; F3, Judgment, critical thinking, open-mindedness; F4, Ingenuity, originality, practical intelligence; F5, Perspective; **D2 = Emotional strength (Courage):** F6, Bravery; F7, Perspective and diligence; F8, Integrity, honesty, authenticity; F9, Vitality and zest; **D3 = interpersonal strength (Humanity):** F10, Love, ability to love and be loved; F11, Kindness, friendliness, generosity; F12, Emotional, personal, and social intelligence; **D4 = Civic strength (Justice):** F13, Citizenship, social responsibility, loyalty, teamwork; F14, Sense of justice, fairness; F15, Leadership; **D5 = Strengths that protect against excess (Temperance):** F16, Ability to forgive, mercy; F17, Modesty, humility; F18, Prudence, discretion, caution; F19, Self-control, self-regulation; **D6 = Strength from the meaning of life (Transcendence):** F20, Appreciation for beauty and excellence, capacity for wonder; F21, Gratitude; F22, Hope, optimism, future-mindedness; F23, Sense of humor; F24, Spirituality, religious sense. **PV = Predictors Variables**. Bold values indicate predictor variables. *p < 0.05; ***p < 0.01; ***p < 0.001.

### Structural and Mediational Prediction Model of Character Strengths, Resilience, and Flourishing

#### Model Testing

Four structural predictive models were tested. The first referred to personal strengths predicting resilience. The second model tested resilience as a predictor of flourishing. The third model tested prediction between resilience and flourishing and the fourth, tested strengths and resilience combined, as joint predictors of flourishing (Table 4). As shown in Table 4, the data fit best to the fourth model as hypothesis 2 stated.

**TABLE 4:** Models of structural linear results.

Chi^2^	FG	CH/df	SRMR	*p <*	NFI	RFI	IFI	TLI	CFI	RMSEA	HOELT.	*p* < 0.05; *p* < 0.01
(1) 120,687	34	3,55	0.09	0.000	0.898	0.835	0.924	0.875	0.923	0.098	0.108	0.125
(2) 75,147	9	8,35	0.10	0.000	0.910	0.789	0.920	0.810	0.918	0.062	0.421	0.522
(3) 106,774	9	11,864	0.12	0.000	0.958	0.901	0.961	0.909	0.961	0.075	0.303	0.338
(4) 256,454	52	4,932	0.05	0.000	0.951	0.954	0.956	0.955	0.966	0.047	0.500	0.526

*Model 1: Strengths, Resilience; Model 2: Strengths, Flourishing; Model 3: Resilience, Flourishing; Model 4: Strengths, Resilience, Flourishing.*

#### Direct Effect

**FIGURE 2:**
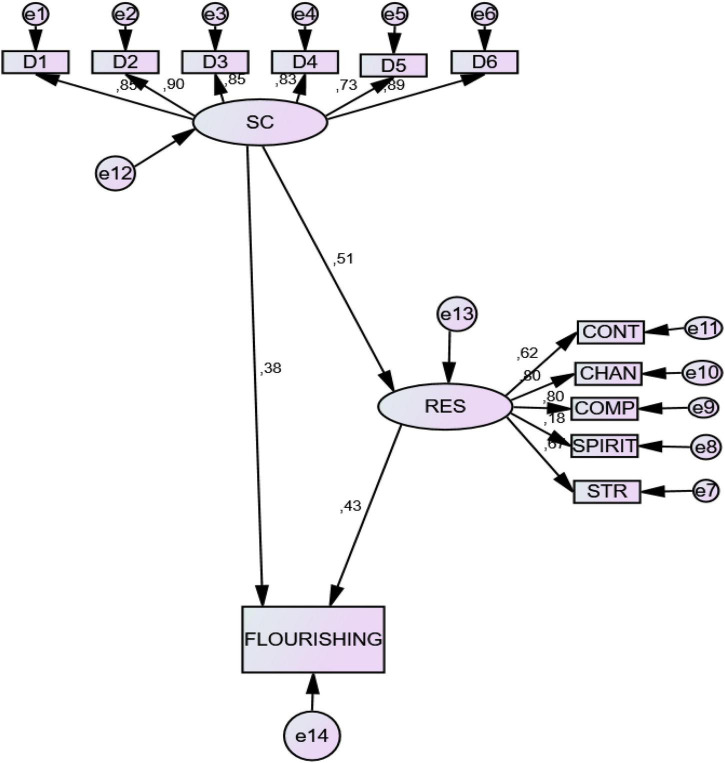
Structural predictive model of Character Strengths, Resilience, and Flourishing. D1, cognitive strength (Wisdom); D2, emotional strength (Courage); D3, interpersonal strength (Humanity); D4, Civic strength (Justice); D5, strengths that protect against excess (Temperance); D6, strength from the meaning of life (Transcendence). STR, Gestion of Stress; SPIRIT, Spirituality; COMPT, Tenacity; CHAN, Adaptation to change; CONT, Self-Control.

Direct statistic effects showed *character strengths* to be significant predictors of resilience (0.51) and flourishing (0.43), and resilience was also a predictor of flourishing (0.60) (see Table 5 and Figure 2).

**TABLE 5:** Total, direct, and indirect effects of the variables under study, and 95% bootstrap confidence intervals (CI).

Predictive variable	Criterion variable	Total effect	CI (95%)	Direct effect	CI (95%)	Indirect effect	CI (95%)	Results effects	CI (95%)
CS - >	Resilience	0.51	[0.39, 0.74]	0.51	[0.39, 0.74]	0.00	[−0.03, 0.02]	Direct only	[0.39, 0.74]
R - >	Fluorishing	0.43	[0.37, 0.54]	0.43	[0.37, 0.54]	0.00	[−0.03, 0.02]	Direct only	[0.37, 0.54]
CS- >	Fluorishing	0.60	[0.41, 0.76]	0.38	[.23, 0.48]	0.22	[0.16, 0.34]	Partial mediation	[0.16, 0.34]
CS- >	Stress	0.34	[0.32, 0.47]	0.00	[−0.04, 0.12]	0.34	[0.26, 0.48]	Full mediation	[0.26, 0.48]
CS- >	Spiritualily	0.09	[0.02, 0.14]	0.00	[−0.03, 0.09]	0.09	[0.02, 0.14]	Full mediation	[0.02, 0.14]
CS- >	Tenacity	0.42	[0.32, 0.57]	0.00	[−0.04, -0.08]	0.42	[0.32, 0.57]	Full mediation	[0.32, 0.57]
CS- >	Change	0.41	[0.20, 0.38]	0.00	[−0.03, 0.04]	0.41	[0.20, 0.38]	Full mediation	[0.20, 0.38]
CS- >	Control	0.32	[0.43, 0.21]	0.00	[−0.07, 0.08]	0.30	[0.43, 0.21]	Full mediation	[0.43, 0.21]

*CS, Character strengths; R, Resilience; CI, confidence interval. Bootstrapping sample size = 643.*

#### Indirect Effect

Additionally, indirect effects were also observed between character strengths and the components of resilience.

#### Mediation Effects

These results demonstrate that character strengths have a significant overall effect on fluorishing (0.60), both direct and indirect. Thus, character strengths have a direct effect on flourishing (0.38) and an indirect effect -through resilience- on flourishing (0.22).

## Discussion

These results partially corroborate findings from previous research, which reveal relationships between character strengths and resilience (Chung, 2008) bringing to light the importance of those strengths.

### Character Strengths and Resilience

Regarding *Hypothesis 1*, which studies the positive linear prediction of strengths on resilience, results uphold that character strengths are foundational to resilient behavior, by showing how character strengths, as a multidimensional construct, significantly, and jointly predict resilience, as prior evidence had founded (Niemiec, 2020; Seale et al., 2020). Thus, the essential value of the present study lies in uncovering a more precise specification of this relationship, from a large sample of Spanish students. These results reveal how each factor of resilience was predicted by one or more character strengths. Specifically, emotional strength (D2) was the strongest predictor of resilient tenacity; cognitive strength (D1) enabled adaption to change; emotional strength (D2) and interpersonal strength (D3) positively predicted control, but the opposite occurred with the strength of temperance (D5); and finally, emotional strength (D2) predicted resilient spirituality. Although with less statistical power, these dimensions also predicted flourishing, confirming previous research (Park and Peterson, 2009).

The factors of character strengths also proved their predictive value for various components of resilience and flourishing. Referring to specific factors of resilience, F9 (vitality and zest) and F7 (perseverance) significantly predicted resilient tenacity. These results suggest a similar behavioral component between the two psychological constructs, but with factorial independence (Martínez-Martí and Ruch, 2017).

Factors F12 (emotional, personal and social intelligence) and F9 (bravery) acted as positive predictors of stress tolerance, whereas F13 (citizenship, social responsibility, loyalty, teamwork) was a negative predictor. This result concurs with abundant evidence that has established emotional intelligence as a protective factor against stress (Elshall et al., 2020; Trigueros et al., 2020). However, this potentially contradictory relationship between citizenship and resilience would need further analysis in future research. Factors F12 (emotional intelligence) and F15 (leadership) positively predicted adaptation to change, while F17 (modesty, humility), F18 (prudence, discretion, caution) and F24 (spirituality, faith, religious sense) were negative predictors. This result is of great interest because it shows that, there is strong likelihood that personal strengths also depend on the circumstance in which one lives. Prudence can also be expected to protect against inappropriate decisions or changes (Reeves et al., 2014).

The resilience factor with the greatest number of predictive character strengths was perceived control, predicted by F9, F14, F10, F2, and F7 (positively) and by F1, F11, F20, F22, and F17 (negatively). These results seem to concur with evidence that personality or character factors predict resilience (Goodman et al., 2017). It is also of interest that factors F21 (gratitude) and F19 (self-control/self-regulation) were positive predictors of spirituality, concurring with previous studies on the relationship between self-regulation and spirituality (Desmond et al., 2013; Boyatzis et al., 2021). However, F24 (spirituality, religious sense) did not predict resilient spirituality, which would suggest that the psychological constructs that are being assessed are somewhat distinct.

One noteworthy result is the negative prediction found between temperance (character strength) and adaptation to change (resilience). This result might indicate that excessive regulation and control, in situations of change, would hinder good adjustment, because of perceived loss of control (Koltai and Stuckler, 2020), which could become deregulatory behavior (de la Fuente-Arias, 2017). Nonetheless, this question should be further studied in future research.

### Strengths, Resilience, and Flourishing

*Hypothesis 2* establishes the combination of character strengths and resilience in a significant, positive relationship with flourishing. The results suggest that the behavioral components inherent in the construct of character strengths predict resilience, and jointly, these two constructs predict flourishing. In short, they predict wellbeing, with similar elements present in both psychological constructs (Chung, 2008; Martínez-Martí and Ruch, 2017; Karris-Bachik et al., 2020). Nonetheless, independence among the three constructs is also clearly shown. Previous research had produced evidence for some of these results. For example, a predictive relationship between strengths and wellbeing had been demonstrated, as well as positivity (Botha, 2020; Wagner et al., 2020). Resilience has consistently appeared as a predictor of wellbeing (Harms et al., 2018). In fact, the resilience variable has appeared as a mediating variable in the relationship between strengths and flourishing; this mediating effect is consistent with other previous work (de la Fuente et al., 2021b).

### Limitations and Future Research

Despite the consistent results, this investigation has several limitations. On the one hand, it should be replicated in broader samples, to compensate for the gender imbalance which is typical of the university samples used here. On the other hand, factor invariance in clinical samples should also be verified. Only in that case can these results be applicable to such samples. Lastly, it remains to be established whether these relationships are generalizable to other populations or areas such as leadership styles (Abbas et al., 2020b), the implementation of coping strategies in the digital age (Abbas et al., 2020a) or coping strategies of rural students at urban universities (Ali et al., 2021).

### Implications for Psycho-Educational Intervention

According to Seligman and Csikszentmihalyi (2000) the field of positive psychology, at the subject level, is about valued subjective experiences: wellbeing, contentment, and satisfaction (in the past); hope and optimism (for the future); and flow and happiness (in the present) (2). At the individual level, it is about positive individual traits: the capacity for love and vocation, courage, interpersonal skill, aesthetic sensibility, perseverance, forgiveness, originality, future mindedness, spirituality, high talent, and wisdom. At the group level, it is about the civic virtues and the institutions that move individuals toward better citizenship: responsibility, nurturance, altruism, civility, moderation, tolerance, and work ethic (Seligman and Csikszentmihalyi, 2000, p. 5).

The present study at the individual level supports the importance of, and a connection between the three constructs analyzed here. For this reason, the university academic curriculum would benefit from inclusion of these three variables, in order to ensure that students receive whole-person training. The importance of academic intervention in improving personal strengths has already been documented (Vargas and González-Torres, 2009; Lavy, 2019). The formation of university students in the role of character strengths, as an essential psychological tool, for the achievement of psychological wellbeing and fluorishing, through the cultivation of resilience, is very important in our university system. Even more so, at a time when hedonic wellbeing proliferates in our university classrooms, as the only way to achieve personal wellbeing.

## Conclusion

These findings have made it possible to corroborate in great detail how the distinct character strength dimensions allow the resilience dimensions in a sample of Spanish students to be predicted. In this sense, the present study showed that: (a) personal strengths can also depend on contextual circumstances; (b) prudence protects against risky decisions; (c) personality or character factors predict resilience; (d) gratitude and self-regulation predict positively spirituality; and (e) the perception of loss of control in situations of change together with excessive regulation and external control hinder the adequate adaptation to new circumstances. Finally, it was established that strengths and resilience present a significant positive predictive effect on flourishing, although clearly denoting the interdependence between the three constructs.

## Data Availability Statement

The raw data supporting the conclusions of this article will be made available by the authors, without undue reservation.

## Ethics Statement

The studies involving human participants were reviewed and approved by Comité de Ética de la Investigación, University of Navarra. The patients/participants provided their written informed consent to participate in this study.

## Author Contributions

JF, BU, EL, and MG-T contributed to the conceptualization, data curation, formal analysis, investigation, methodology, and writing—original draft. JF contributed to the design, supervisión, data curation, formal analysis, methodology, and was responsible for the overall content as the guarantor. BU, EL, MG-T, RA-G, and AB contributed to investigation, methodology, and resources. All authors contributed to reviewing and editing the final manuscript and to the article and approved the submitted version.

## Conflict of Interest

The authors declare that the research was conducted in the absence of any commercial or financial relationships that could be construed as a potential conflict of interest.

## Publisher’s Note

All claims expressed in this article are solely those of the authors and do not necessarily represent those of their affiliated organizations, or those of the publisher, the editors and the reviewers. Any product that may be evaluated in this article, or claim that may be made by its manufacturer, is not guaranteed or endorsed by the publisher.
